# Parenteral Ready-to-Use Fixed-Dose Combinations Including NSAIDs with Paracetamol or Metamizole for Multimodal Analgesia—Approved Products and Challenges

**DOI:** 10.3390/ph16081084

**Published:** 2023-07-31

**Authors:** Fernando Silva, Gustavo Costa, Francisco Veiga, Catarina Cardoso, Ana Cláudia Paiva-Santos

**Affiliations:** 1 Department of Pharmaceutical Technology, Faculty of Pharmacy, University of Coimbra, 3000-548 Coimbra, Portugal; 2 REQUIMTE/LAQV, Group of Pharmaceutical Technology, Faculty of Pharmacy, University of Coimbra, 3000-548 Coimbra, Portugal; 3 Coimbra Institute for Clinical and Biomedical Research (iCBR), Faculty of Medicine, University of Coimbra, 3000-548 Coimbra, Portugal; 4 Center for Innovative Biomedicine and Biotechnology (CIBB), Faculty of Medicine, University of Coimbra, 3000-548 Coimbra, Portugal; 5 Laboratory of Pharmacognosy, Faculty of Pharmacy, University of Coimbra, Azinhaga de Santa Comba, Pólo das Ciências da Saúde, 3000-548 Coimbra, Portugal; 6 Laboratórios Basi, Parque Industrial Manuel Lourenço Ferreira, Lote 15, 3450-232 Mortágua, Portugal

**Keywords:** fixed-dose combination, NSAID, non-opioid analgesic, ready-to-use, parenteral, multimodal analgesia, pain

## Abstract

The combination of non-steroidal anti-inflammatory drugs (NSAIDs) with non-opioid analgesics is common in clinical practice for the treatment of acute painful conditions like post-operative and post-traumatic pain. Despite the satisfactory results achieved by oral analgesics, parenteral analgesia remains a key tool in the treatment of painful conditions when the enteral routes of administration are inconvenient. Parenteral ready-to-use fixed-dose combinations of non-opioid analgesics combinations, including NSAIDs and paracetamol or metamizole, could play a central role in the treatment of painful conditions by combining the advantages of multimodal and parenteral analgesia in a single formulation. Surprisingly, only in 2020, a parenteral ready-to-use fixed-dose combination of ibuprofen/paracetamol was launched to the market. This review aims to investigate the current availability of combinations of NSAIDs with paracetamol or metamizole in both European and American markets, and how the combination of such drugs could play a central role in a multimodal analgesia strategy. Also, we explored how the parenteral formulations of NSAIDs, paracetamol, and metamizole could serve as starting elements for the development of new parenteral ready-to-use fixed-dose combinations. We concluded that, despite the well-recognized utility of combining NSAIDs with paracetamol or metamizole, several randomized clinical trial studies demonstrate no clear advantages concerning their efficacy and safety. Future clinical trials specifically designed to assess the efficacy and safety of pre-formulated fixed-dose combinations are required to generate solid evidence about their clinical advantages.

## 1. Introduction

Severe acute pain remains a major problem associated with trauma-related injuries and surgery procedures. Acute pain persists as an unavoidable outcome and all efforts should be performed to manage the impact of painful conditions in a patient’s recovery [1,2,3]. Besides the emotional impact, the suffering induced by untreated acute pain can result in physical problems such as myocardial ischemia, impaired wound healing, delayed gastrointestinal motility, and poor respiratory effort [3,4]. Also, poor respiratory effort can result in atelectasis, hypercarbia, or hypoxemia and thus contribute to a higher incidence of post-operative pneumonia [3]. 

Among analgesics, opioids are considered the “gold standard” for the treatment of painful conditions after surgery procedures. Nevertheless, they offer only moderate efficacy in relieving pain during movement while having relevant side effects, such as nausea, vomiting, and a high risk of addiction [4,5,6]. 

Multimodal pain management guidelines have proposed the implementation of non-opioid analgesic procedures aiming to avoid the use of opioid substances in monotherapy or at least reduce the doses used for acute pain treatment [7]. 

The combination of non-opioid analgesics, mainly paracetamol (or acetaminophen) with non-steroidal anti-inflammatory drugs (NSAIDs), is one of the most reported multimodal approaches used in clinical practice [2,8,9,10]. The guidelines for acute pain management issued by the American Society of Anesthesiologists (ASA) recognized the effective and well-established use of combined paracetamol and NSAIDs for several types of pain [2,3,5,9,11,12]. Also, the Enhanced Recovery After Surgery (ERAS) Society guidelines recommend a multimodal approach through the administration of paracetamol and NSAIDs in combination [13,14]. This kind of combination has been a central focus of ERAS protocols since it reduces the need for opioid drugs, diminishing simultaneously painful and inflammatory conditions [2,7,9,11,15]. 

In general, fixed-dose combinations (FDCs) offer the opportunity to improve the therapeutic response in people where monotherapy approaches have failed [16]. Also, FDCs could contribute markedly to pain management in low-income countries where the availability of opioid drugs is extremely limited and non-opioid analgesics are generally cheap. Indeed, in 2011, 89% of globally available opioids were consumed only by the USA (United States of America), Canada, the UK (United Kingdom), and Australia [1].

Ibuprofen/paracetamol FDC in tablets are available in the US and some European markets as over-the-counter (OTC) products. Also, in some European countries, but not in the US, ibuprofen/paracetamol FDCs are approved for intravenous (IV) administration.

Since the parenteral administration of analgesic entities allows for a rapid onset of action and pain management in patients that are unable to intake oral formulations [3,7], parenteral FDCs of non-opioid analgesics composed of NSAIDs may play a decisive role in the treatment of acute pain due to the possibility of simultaneously solving some limitations related to monotherapy regimens [16]. 

This work aims to describe the role of ready-to-use parenteral FDCs composed of NSAIDs with paracetamol or metamizole in the treatment of acute painful conditions and their increased value when used in multimodal analgesia regimens. Also, we explored the available literature to study the clinical evidence regarding the efficacy and safety of combining NSAIDs with paracetamol or metamizole and analyze the scientific results reporting the ability of these combinations to offer higher efficient analgesic capability when parenterally administered.

## 2. Multimodal and Parenteral Analgesia

### 2.1. Multimodal Analgesia

Pain pathways are a complex system where a noxious stimulus is converted into a neural signal resulting in the perception of an unpleasant experience. Beyond the negative impact at the emotional level, acute pain deeply challenges patients’ mobilization and may compromise surgery outcomes [10].

Due to the complexity of pain physiology, a single analgesic drug able to completely suppress pain perception remains an impossibility. To accomplish such a task, more effective and satisfying results could be achieved by using multimodal analgesia approaches [2,3,4,10]. 

Multimodal analgesia entails the administration of different pharmacological agents with different mechanisms of action to obtain improved analgesic outcomes than those obtained via single-drug administration. At the same time, a potential synergic relationship between the combined drugs may allow for the reduction of drug doses and thus induce fewer side effects [2,3,12,17]. 

Multimodal analgesia aims to avoid the use of opioids or at least significantly reduce their effective doses [13]. At the same time, it is related to the early mobilization and discharge of patients, fewer readmission rates, and improved patient satisfaction [17]. Also, many works have reported multimodal analgesia as a fundamental approach to minimizing unnecessary opioid use and a relevant tool to manage the risk of opioid addiction [3,17]. In the clinical context, multimodal analgesia has been recommended in numerous pain management guidelines issued by several renowned medical institutions such as the ASA, American Academy of Pain Medicine (AAPM), Orthopaedic Trauma Association (OTA), and Doctors Without Borders (MSF), among others. For instance, in 2015, a partnership between the AAPM, ASA, US Department of Veterans Health Administration, and Department of Defense resulted in the definition of new clinical practice guidelines for post-operative pain management [18]. Based on these guidelines, the multimodal approach using NSAID combinations with paracetamol is presented as a strong recommendation with high-quality evidence [18]. Additionally, over the last few years, ERAS protocols are becoming the benchmark standards for enhancing post-operative recovery [14]. In these guidelines, multimodal analgesia has now established itself as the “gold standard” of perioperative analgesic care due its prominent implementation as an essential component in pain treatment [14]. 

Several classes of drugs can be used in combination. Non-opioid analgesics (including NSAIDs), N-methyl-D-aspartate (NMDA)-receptor antagonists, gabapentinoids, α_2_-receptor agonists, local anesthetics, and selective serotonin reuptake inhibitors (SSRIs) are some examples of drug classes that are being used as analgesic entities [2,4,7,10,12,14]. Notably, some of these classes of drugs were originally developed and approved not to treat painful conditions but to treat other pathologic conditions such as depression, Alzheimer’s disease, epilepsy, and hypertension. The analgesic activity of this heterogeneous group of pharmacological entities is explained by the physiology of pain and its mechanisms of transmission [17]. 

Pain can be divided into two major types: nociceptive and neuropathic. The first one has physiological functions and is related to noxious stimuli and tissue damage. The second one is precepted when the somatosensory system itself is damaged and is recognized as a pathological condition [17,19]. Based on the type, location, and patient perception of pain, one or more pain pathways can be targeted using analgesic combinations [17].

### 2.2. Parenteral Analgesia

Parenteral analgesics allow for the administration of drugs when more convenient routes are not clinically available [3,7]. The most common parenteral routes of administration are IV, intramuscular (IM), and subcutaneous (SC) [20]. IV analgesia in one of the most common approaches used in the management of acute pain induced by surgical procedures [14,21]. Also, the IM and SC routes are commonly employed for the treatment of acute pain; however, after surgery, they should not be considered first-line options since the poor perfusion and delayed distribution of the drugs is a risky possibility and can result in the occurrence of inadequate analgesia or the late occurrence of side effects [20]. Although IM and SC formulations may be appropriate for patients without IV access and with the oral route unavailable, IV administration is believed to provide the fastest relief [3,4,22,23,24,25]. However, when compared to other routes of administration, the costs related to the use of IV forms are higher and with no benefits in terms of pain control [20].

Despite the evident advantages of the use of parenteral analgesics, their development and manufacture can be challenging and expensive [26]. For instance, the poor water solubility of ibuprofen and diclofenac can result in the development of unstable products; post-administration crystallization may lead to the formation of aggregates and emboli in blood vessels [26,27]. In contrast, metamizole is very soluble in aqueous solutions but stability issues may also be raised [28]. 

Despite the large number of non-opioid analgesics available on the market, there is yet a lack of options concerning parenteral solutions. Until 2020 in the US, only parenteral formulations of paracetamol, ibuprofen, and ketorolac had been approved for post-operative pain management in monotherapy regimens [7]. In 2020, an IV formulation of meloxicam was approved by the US Food and Drug Administration (FDA), but in December 2022, its commercialization was discontinued since, according to Baudax Bio (market applicant), “despite having distinct benefits as the first and only once-daily non-opioid IV analgesic, market conditions are not favorable for the introduction and commercialization of a new pain management product in the hospital market” [29]. Concerning the European market, the number of approved parenteral non-opioid analgesics is larger; however, their availability is different between sovereign countries (Table 1).

The parenteral administration of some analgesic drugs can impact the pharmacologic profile (i.e., pharmacokinetics and pharmacodynamics). For instance, the pharmacologic activity of paracetamol depends on its route of administration. When administered orally, paracetamol will suffer first-pass metabolism and be converted into p-aminophenol in the liver and then into N-(4-Hydroxyphenyl) arachidonylamide (AM404) in the brain. The AM404 is a potent agonist of the transient receptor potential vanilloid type 1 (TRPV1), a low-affinity ligand of the cannabinoid receptor type 1 (CB_1_), an anandamide membrane transporter blocker, and a cyclooxygenase (COX) inhibitor [30]. When paracetamol is taken intravenously, it is spared from the first-pass process and the biosynthesis of AM404 is less extensive [31]. Also, intravenously, the maximum plasma concentration of paracetamol is greater and achieved earlier. Since paracetamol enters the central nervous system (CNS) through passive diffusion, the gradient concentration between the plasma and CNS tissues is a very important factor for its infiltration into the CNS [23,24].

In contrast to paracetamol, metamizole pharmacology seems not to be impacted by the route of administration since the bioconversion of metamizole in their active metabolite occurs extensively in the stomach and plasma [32,33].

Concerning NSAIDs, oral administration is as efficacious as intravenous intervention since their oral bioavailability is commonly very high [34,35]. The most evident advantage of the intravenous administration of NSAIDs is their fast onset when compared with enteric routes [34,35]. For instance, the maximum plasma concentration achieved after the IV administration of ibuprofen is twice as high as the concentration achieved through oral routes and earlier administration (IV: 0.11 h vs. oral: 1.5 h) [36]. Since the mechanism of action and the pharmacology of NSAIDs are independent of the route of administration, an earlier achievement of maximum plasma concentration commonly results in a fast onset [21,37,38].

#### 2.2.1. Paracetamol-Based Parenteral Formulations

The mechanism of action of paracetamol is not completely understood. However, it is probably mediated through COX-inhibition in the central nervous system [13,39,40]. Research has suggested that paracetamol acts as an inhibitor, particularly for COX inhibitor-3, a COX-1 isoenzyme mainly expressed in the central nervous system [13,39]. Also, its analgesic mechanism could be related to the activation of the descending serotonergic pathway, the indirect activation of CB_1_ receptors, and the inhibition of nitric oxide pathways in the central nervous system [3,17].

The paracetamol metabolite AM404 also plays a very relevant role concerning analgesic activity. Like paracetamol, its mechanism of action is not completely understood; however, it seems to act in the blockade of the neuronal uptake of anandamide and neuronal sodium channels [40].

Peripherally, although paracetamol exerts COX-1 and 2 inhibition in in vitro assays, the expected anti-inflammatory activity is not observed in vivo since the high concentrations of peroxide in inflamed tissues hinder the paracetamol activity [3].

The IV administration of paracetamol and NSAIDs allows for a faster effect onset and greater peak plasma concentration compared to oral administration [4,7,40]. Additionally, IV administration seems to be less hepatotoxic than the oral route due to the absence of first-pass phenomena [4]. Remarkably, no evidence of hepatotoxicity was observed after administration of 5 g of paracetamol via IV across 24 h in healthy subjects, a higher dosage than recommended [4].

Paracetamol is the most common analgesic used in multimodal analgesia since its administration is generally well tolerated, with a minimal side effect profile [14]. In Europe and the US, IV formulations of paracetamol have been available on the market since late 2002 and November 2010, respectively [4,25,31]. The common formulation is 1000 mg/100 mL and the brands available are listed on the FDA website and on the list of nationally authorized medicinal products (PSUSA/00002311/201705) issued by the European Medicines Agency (EMA) [41,42].

Despite the numerous IV formulations of paracetamol available on the market, their development was always challenging due to the poor stability of paracetamol [43]. Indeed, during degradation, paracetamol is converted by hydrolysis into 4-aminophenol, which is rapidly converted into the hepatotoxic substance N-acetyl-*p*-benzoquinone imine (NAPQI). At the same time, oxidation reactions occur, producing degradation products. To avoid degradation, the synthesis of active pharmaceutical ingredients (APIs) and the manufacturing of a parenteral finished product should be performed under optimal pH values (from 5 to 6) and low oxygen media (bubbling nitrogen) [43].

#### 2.2.2. Metamizole-Based Parenteral Formulations

Metamizole (or dipyrone) is a non-acidic analgesic like paracetamol but belongs to the group of phenazones [44]. Like paracetamol, metamizole is a common analgesic and antipyretic drug but with low anti-inflammatory activity. It is generally well tolerated and can treat several painful conditions including post-operative pain, headaches, migraines, neuropathic pain, cholic pain, and cancer pain [44,45,46,47,48]. For post-surgery purposes, metamizole is a more effective analgesic than paracetamol and at least as effective as NSAIDs [47].

As monotherapy and due to its presumably favorable safety profile, metamizole is preferred over NSAIDs [44]. However, metamizole has been related to cases of severe neutropenia and agranulocytosis (myelotoxicity) and, due to this fact, it was banned from the market in some countries such as the US, the UK, Sweden, Canada, Australia, Norway, and India [46,47,48,49,50,51,52]. Nevertheless, metamizole is still available in some European and South American countries [44,47,48,49]. Indeed, the incidence of metamizole-induced myelotoxicity is controversial and varies widely between studies. Yet, some works point to a risk of approximately 1:1602 and a relative risk of 3.03 [46,53]. Clinical studies with low enrolment and some other limitations like ethnicity factors could explain the significant differences reported by authors about the incidence of adverse events associated with metamizole [51,52,54].

The mechanisms of action are not completely understood; however, in the literature, metamizole is presented as a prodrug, while its metabolites act at both peripheral and central levels [44,47,48]. 4-*N*-methyl-aminoantipyrine (4-MAA) is the main metabolite in plasma [47,55,56]. This metabolite is biosynthesized non-enzymatically from metamizole in the gastrointestinal tract and its extensive absorption results in a bioavailability close to 100% [47,55,56].

Some authors have reported the inhibition of COX activity to be the main mechanism of action of metamizole. However, metamizole metabolites seem to directly block the hyperalgesia induced by prostaglandin E2 (PGE2) and isoprenaline through a COX-independent mechanism [44,47,48]. Interestingly, like paracetamol, metamizole presents COX inhibition activity in vitro (mainly COX-2 activity) but exhibits a weak anti-inflammatory ability and low gastrointestinal toxicity in humans [48]. Recently, Gomes F. et al. [56] reported that metamizole directly blocks nociceptor sensitization via the activation of the NO signaling pathway. The same authors hypothesized that metamizole promotes the engagement of the PI3Kγ/AKT/nNOS/cGMP pathway, which results in the hyperpolarization of the primary sensory neuron terminals and decreases neuronal excitability [56]. Also, Gonçalves do Santos G. et al. [57] have reported that the 4-MAA anti-hyperalgesic effect depends on κ-opioid receptor activation, acting as a morphine-like drug [52,56,58,59]. Despite all of this, metamizole is yet classified incorrectly as an NSAID by some authors [50].

The mechanism responsible for agranulocytosis is not fully understood; however, some authors excluded a direct toxic effect of metamizole by pointing to an immunoallergic reaction as a possible hypothesis [50,53]. In the presence of heme iron, 4-MAA forms reactive electrophilic entities that are toxic for granulocyte precursors. This can occur mainly when there is a depletion of the cellular adenosine triphosphate (ATP) pool [47].

In contrast to paracetamol, metamizole is very soluble in water; however, it is chemically unstable [28,60]. Since metamizole is hydrolyzed rapidly and non-enzymatically to its active metabolite, 4-MAA, its stability verified in commercial liquid formulations is achieved using high concentrations of metamizole. The concentration is the major factor in the hydrolysis of metamizole and thus increasing the concentration of metamizole decreases the hydrolysis rate [61].

In the European market, metamizole for parenteral use is commonly available in vials of 500 mg/1 mL, 1 g/2 mL, or 2 g/5 mL for IV or IM use. The list of products available in European countries can be found on the list of nationally authorized medicinal products (PSUSA/00001997/202103) issued by the EMA.

#### 2.2.3. NSAID-Based Parenteral Formulations

NSAIDs are the most consumed drugs worldwide and the most common option available for the treatment of mild-to-moderate inflammatory pain without an additive effect [62]. The main and better-understood mechanism of action of NSAIDs is the inhibition of peripheral COX-1 and COX-2, two enzymes that play a crucial function in the production of pro-inflammatory prostaglandins [2,39]. Other additional mechanisms in the central nervous system are being proposed based on in vitro and animal experiments, without clear evidence of their occurrence in humans [11]. A central action by NSAIDs in humans is unlikely, or at least negligible, due to the pharmacokinetic profile of NSAIDs since their low distribution volume may reveal the slow or inadequate penetration of NSAIDs in the CNS [11].

As in the case of paracetamol, NSAIDs are also a key element in multimodal analgesia since they can provide superior analgesia with opioid-sparing and with fewer side effects such as nausea, vomiting, and unwanted sedation [14]. As cited previously, despite the large number of NSAIDs approved in the US and Europe, they are not all available in parenteral formulations. Currently, there is a lack of IV NSAIDs available on the market and there is a need for the development of new IV NSAID-based formulations [29]. Unfortunately, the very low market quote (February 2020–December 2022) and recent IV meloxicam discontinuation (Anjeso) from the US market may indicate that there is not a market available for new parenteral pain killers.

Table 1 presents all the parenteral NSAIDs available in the US and Europe. The number of NSAIDs authorized in Europe is larger; however, not all USA-approved products are also approved in Europe. Ibuprofen and ketoprofen are available for parenteral administration in Europe and the US. However, some differences still exist. Concerning ibuprofen, in the US, ampoules and flasks with 800 mg/8 mL and 800 mg/200 mL are available, while in Europe, only large-volume formulations exist. The maximum dose of ibuprofen is higher in the US (800 mg) than in Europe (600 mg). Also, small differences are observed when comparing ketorolac formulations available in the US and Europe; however, in both cases, the drug can be administered via IV or IM (Table 1).

Aspegic^®^ (acetylsalicylate), Xefo^®^ (lornoxicam), Neo-Indusix^®^ (tenoxicam), and Liometacen^®^ (indomethacin) are only available in Europe and are presented as freeze-dried products. The freeze-drying process is technically challenging, expensive, and yields fragile and hygroscopic products. However, in the case of less soluble drugs, such as indomethacin, tenoxicam, and lornoxicam, it can avoid the undesired crystallization of drugs during their storage [63,64,65]. Concerning acetylsalicylate formulation, although its salts are commonly soluble in water, freeze-drying is useful due to the extensive hydrolysis of salicylic salts in aqueous media [66].

Most NSAIDs share the same therapeutic indications; however, some of them are recommended predominantly for specific painful conditions. According to Table 1, the listed NSAIDs may be sorted into three groups: NSAIDs indicated predominantly for the treatment of musculoskeletal-system-related pain, NSAIDs indicated for the treatment of postoperative pain/post-traumatic-related pain, and NSAIDs indicated for the treatment of unclear painful conditions. Nevertheless, in the case of piroxicam and meloxicam, their therapeutic indications seem to be more restricted since in their Summary of Product Characteristics (SPCs), the first-line use of these products is discouraged due to their safety profiles [67,68].

All conventional NSAIDs are weak acids and, when they are taken orally, their molecules adopt uncharged conformations due to the strongly acidic environment of the stomach [69,70]. The uncharged state of the molecules allows for their rapid absorption through the gastric surface epithelium [69]. Since NSAIDs present generally high bioavailability after oral administration, the parenteral route is only recommended when less invasive routes are not available [34,35,71]. In contrast with paracetamol, where parenteral administration reduces hepatotoxicity, there is not clear evidence of the superior efficacy and safety of NSAIDs parenterally administered [35,71].

**Table 1:** Parenteral-NSAIDs-based medicines approved in the USA and Europe.

NSAID	Formulations	Route of Administration	Indications	Brands ^a^	Countries with Marketing Authorization ^d^
Ibuprofen	800 mg/8 mL 800 mg/200 mL	IV	Management of mild-to-moderate pain and moderate-to-severe pain in adults. Also, it is indicated for the reduction of fever in adults [72].	Caldolor^®^	US
Ketorolac	15 mg/1 mL 30 mg/1 mL	IV/IM	Short-term management of moderate-to-severe acute pain, including pain following operative procedures [73].	Toradol^® b^	US
Acetylsalicylate	500 mg/5 mL (freeze-dried)	IM/IV	Symptomatic treatment of pain in rheumatology, traumatology, oncology, surgery and anaesthesiology, post-operatively, and in preparation for exams. Also used in the symptomatic treatment of fever [74].	Aspegic^®^	BE, HU, and PT
Dexketoprofen	50 mg/2 mL 25 mg/2 mL	IM/IV	Symptomatic treatment of acute pain of moderate-to-severe intensity when oral administration is not appropriate, such as post-operative pain, renal colic, and lower back pain [75,76].	Ketesse^®^ Keral^®^ Auxilen^®^ Dekenor^®^ Morsadex^®^	DE, AT, SK, SI, ES, EE, FI, FR, GR, NL, HU, IE, LV, LT, MT, PL, CZ, and RW
	50 mg/100 mL	IV		Dexketoprofen B. Braun^®^	ES
Diclofenac	75 mg/3 mL ^c^ 50 mg/1 mL 25 mg/1 mL	IV/IM	IM use is effective in acute forms of pain, including renal colic, exacerbations of osteo- and rheumatoid arthritis, acute back pain, acute gout, acute trauma and fractures, and post-operative pain. In IV use, it is indicated for the treatment or prevention of post-operative pain in the hospital setting [77].	Voltaren^®^ Voltarol^®^ Fenil-V^®^ Akis^®^ plus Dicloin^®^ Diclac^®^ Almiral^®^	DE, AT, BE, BG, SK, SI, ES, EE, FR, FI, GR, NL, HU, IE, IT, LV, LT, MT, PL, PT, GB, CZ, RW, and SE
Etofenamate	1000 mg/2 mL	IM	Indicated in painful and acute inflammatory situations in rheumatology, traumatology, and post-operatively [78].	Rheumon^®^ Traumon^®^	DE, AT, GR, HU, PT, and RW
Ibuprofen	600 mg/100 mL ^c^ 400 mg/100 mL 200 mg/50 mL	IV	Indicated in adults for the short-term symptomatic treatment of acute moderate pain and fever. IV route is clinically justified when other routes of administration are not possible [79].	Ibuprofen B. Braun^®^ Solibu^®^	DE, AT, BE, BG, DK, SK, SI, ES, EE, FI, NL, HU, IE, LV, LT, PL, PT, GB, CZ, RW, and SE
Indomethacin	50 mg/2 mL 25 mg/2 mL (freeze-dried)	IV	Indicated to reduce (acute) pain due to inflammation of the muscles and muscle joints (musculoskeletal system) [80].	Liometacen^®^	IT
Ketoprofen	100 mg/2 mL ^c^	IM	Indicated for rheumatoid arthritis, osteoarthritis ankylosing spondylitis, and acute episodes of gout. The injectable form is especially indicated for the treatment of acute attacks with a predominance of pain [81].	Profenid^®^ Rofenid^®^ Ketonal^®^ Orudis^®^	BE, SK, SI, ES, FR, IT, LV, LT, PL, PT, CZ, and RW
Ketorolac	50 mg/5 mL 30 mg/1 mL ^c^ 10 mg/1 mL	IM/IV	It is indicated for the short-term management of moderate-to-severe acute post-operative pain. Treatment should only be initiated in hospitals. The maximum duration of treatment is two days [82].	Toradol^®^ Taradyl^®^	BE, DK, ES, EE, FI, GR, IS, IT, LV, LT, PT, GB, RW, and SE
Lornoxicam	8 mg/2 mL (freeze-dried)	IM/IV	Short-term relief of acute mild-to-moderate pain [83].	Xefo^®^	SK, GR, HU, and RW
Meloxicam	15 mg/1.5 mL	IM	Short-term treatment of symptomatic acute exacerbations of rheumatoid arthritis and ankylosing spondylitis when other routes of administration are not appropriate [68].	Movalis^®^ Melox^®^ Mobic^®^	SK, EE, FR, GR, HU, IT, LV, LT, MT, PL, PT, and RW
Piroxicam	20 mg/1 mL	IM	Symptomatic relief of osteoarthritis, rheumatoid arthritis, and ankylosing spondylitis [67].	Feldene^®^ Flexase^®^	DE, BE, ES, FR, HU, PL, and PT
Tenoxicam	20 mg/3 mL ^c^ (freeze-dried)	IM/IV	Indicated for patients considered unable to take oral tenoxicam for the relief of pain and inflammation in osteoarthritis and rheumatoid arthritis and for the short-term management of acute musculoskeletal disorders including strains, sprains, and other soft-tissue injuries [84].	Neo-Indusix^®^	GR, GB, and RW

Abbreviations: IM—intramuscular, IV—intravenous, IM/IV—intramuscular and intravenous. ^a^ Here are presented only the main brands found during the research. However, in many cases, there are available on the market other products with other brand names. ^b^ According to the data obtained from the FDA website, Toradol^®^ is no longer available in the USA. Instead, generic products are sold in the USA. Toradol^®^ is presented in the table as this is the brand most cited in the literature when ketorolac formulations are mentioned. ^c^ There are available on the market identical products where the drug dosage is the same but the liquid volumes differ slightly. Despite this, the product characteristics are not impacted. ^d^ Based on the information available in the databases consulted on the websites of the Health Authority of each European country. Countries are listed using a two-letter country code.

## 3. Parenteral Fixed-Dose Combinations

The combination of analgesic entities is a common practice in the clinical setting. As reported previously, the complexity of pain physiology does not facilitate the induction of satisfactory analgesia in all painful conditions using a single drug.

When administered in monotherapy, NSAIDs have been related to cardiovascular, gastrointestinal, and renal adverse effects [62]. Unfortunately, even selective COX-2 NSAIDs are related to the occurrence of markable cardiovascular and renal adverse events since the physiologic role of COX-2 in the vascular system and kidneys was only discovered after the development of this NSAID class [62]. Considering that NSAIDs can increase the risk of the occurrence of severe adverse events in a dose-dependent manner, the combination of NSAIDs with paracetamol or metamizole could lead to the development of safer analgesic products with reduced doses of NSAIDs [85,86].

Combinations of NSAIDs with paracetamol have presented a greater antinociceptive effect compared with the respective drugs alone and have a superior morphine-sparing effect when compared with corticosteroids, tramadol, nefopam, corticosteroids, and metamizole alone [5]. This effect could be related to synergic interactions between the dissimilar mechanisms of action [87]. Miranda H. et al. [87] demonstrated the synergic relationship between paracetamol and NSAIDs by isobolographic analysis in mice using the writhing test. The authors concluded that their results validate the clinical use of NSAIDs in combination with paracetamol for the treatment of painful conditions [87].

Considering the significant number of drugs available to be administered in multimodal analgesia, we may assume the pharmaceutical development of numerous analgesic FDCs to be theoretically possible. Instead, in American and European markets, the unique parenteral analgesic FDC approved at this moment is paracetamol with ibuprofen. Below, a complete literature search on the development of parenteral FDCs of NSAIDs with NOAs is presented.

In subchapter 3.3, a literature search for randomized clinical trials (RCTs) testing the utility of combining NSAIDs with paracetamol/propacetamol and metamizole for clinical proposes is reported. However, applying the inclusion and exclusion criteria described there, paracetamol/propacetamol but not metamizole combinations with NSAIDs were found.

### 3.1. Paracetamol-Based Parenteral Fixed-Dose Combinations

Ibuprofen and paracetamol are among the most-consumed analgesics. Usually, they are widely available even without a medical prescription [88]. As previously reported, at this moment, the combination of ibuprofen with paracetamol is the unique parenteral analgesic FDC available on the market. Even when taken orally, this combination was shown to be an effective alternative to opioid-based analgesia [30]. However, there are a limited number of studies assessing the efficacy and safety of parenteral FDCs of ibuprofen/paracetamol [3].

As reported previously, the combination of some NSAIDs with paracetamol or metamizole is widely established in clinical practice. Still, the procedures related to the introduction of ready-to-use FDCs of these drugs in the European and US markets are not well established yet. In 2015, Vale Pharmaceuticals Ltd. (Ireland, United Kingdom) submitted a marketing authorization application to the UK under the three decentralized procedures (DCPs) (UK/H/6034/001/DC, UK/H/6035/001/DC, and UK/H/6176/001/DC) for an oral FDC of ibuprofen (150 mg)/paracetamol (500 mg) film-coated tablets [89]. In this process, the UK was defined as the Reference Member State, and Austria, Germany, Croatia, Ireland, Luxembourg, France, Belgium, the Netherlands, Portugal, and Spain were involved as Concerned Member States. During the process, Germany, France, the Netherlands, and Spain raised major issues regarding efficacy and safety data. According to these countries, the clinical trials presented during the submission were not able to demonstrate the added value of the new oral FDC. Subsequently, the absence of an agreement at the Coordination Group for Mutual Recognition and Decentralized Procedures—Human (CMDh) led this matter to arbitration with the Agency’s Committee for Medicinal Products for Human Use (CHMP). In 2017, the CHMP completed the assessment and reported that the benefits of ibuprofen/paracetamol film-coated tablets outweigh its risks. The CHMP concluded that this combination was more effective than the components individually, while its safety profile was similar [90]. The clinical aspects of this application were supported by five clinical studies sponsored by AFT Pharmaceuticals Ltd. (Auckland, New Zealand) (AFT-MX-1, AFT-MX-3, AFT-MX-4, AFT-MX-6, and AFT-MX-6E) to demonstrate the efficacy and safety of this oral FDC [89,91,92].

In 2020, the IV ready-to-use FDC of ibuprofen (3 mg/mL)/paracetamol (10 mg/mL) of Vale Pharmaceuticals Ltd. (also named Comboval^®^ or Combofusiv^®^) was approved in Europe. According to the Public Assessment report of the product (PAR SE/H/1948/01/DC), this product was developed to extend the therapeutic advantage of the ibuprofen/paracetamol FDC to patients where the IV route is clinically justified [91]. In 2021, the parenteral FDC Maxigesic^®^ of AFT Pharmaceuticals (PAR SE/H/2093/001/DC) was approved [92]. In both applications, in addition to the supportive RCT studies cited in the previous paragraph were considered the clinical studies coded as AFT-MXIV-01, AFT-MXIV-06, and AFT-MXIV-07 [89,90,91,92].

Concerning the North American market, only in 2020, the FDA approved the first oral analgesic FDC (ibuprofen (125 mg)/paracetamol (250 mg)) for the treatment of acute painful conditions [93]. In contrast to the European market, no parenteral combination of ibuprofen/paracetamol had been approved yet in the US. However, the FDA accepted in October 2021 a New Drug Application for the parenteral FDC Maxigesic^®^ IV (also named Combogesic^®^) [94].

The clinical trials presented on the marketing authorization applications were performed by Vale Pharma and AFT Pharmaceuticals to support the safety and efficacy of ibuprofen/paracetamol FDC. A summary of all the clinical trials performed by Vale and AFT was published by Aitken et al. [30]. In total, ten phase I, four phase II/III, and one long-term exposure (phase II) clinical trials were performed [30]. In this work, the authors reported excellent tolerability and safety profiles after the administration of ibuprofen/paracetamol FDC. The FDC provided a greater opioid-sparing effect when compared to paracetamol monotherapy and did not increase the incidence of adverse events [30]. Also, concerning the pharmacokinetics of the combination, studies report an absence of interactions between ibuprofen and paracetamol during concomitant oral or IV administration [88].

More recently, a meta-analysis was performed by Abushanab D. & Al-Badriyeh D. [93] aiming to assess both the efficacy and safety of ibuprofen/paracetamol FDC for the treatment of post-operative pain in adults. In this work, seven double-blind, randomized controlled trials with 2947 participants were included and three FDC dose levels were considered: ibuprofen (75 to 100 mg)/paracetamol (250 mg), ibuprofen (150 to 200 mg)/paracetamol (500 mg), and ibuprofen (292.5 to 400 mg)/paracetamol (975 to 1000 mg) [93]. The authors reported that the ≥ 50% pain relief outcome was better achieved with the FDC compared to a placebo (risk ratio [RR] 2.60, 95% confidence interval [CI] 2.11–3.20, *p* < 0.00001) and reduced the need for rescue medications (RR 0.51, 95% CI 0.37–0.71, *p* < 0.0001). However, the authors reported that the safety outcomes were inconclusive and warned of the need for future studies to confirm its safety and benefits against other marketed analgesics in post-operative pain [93]. With these results, the authors concluded that IBP/APAP FDCs are effective in the treatment of moderate-to-severe pain in adults after surgical procedures [93].

In the previously cited meta-analysis [93], only some clinical studies were selected where an ibuprofen/paracetamol combination was assessed through the administration of FDCs. Nonetheless, there are results of many other clinical trials in the literature where the combination of ibuprofen and paracetamol was performed either with an FDC or separately [23,24,95,96,97,98,99,100,101,102,103,104,105,106,107,108,109,110]. In contrast with the results obtained by Abushanab D. & Al-Badriyeh D. [93], where the administration of ibuprofen/paracetamol FDCs seems to provide generally improved analgesia, no significant or clear advantages of an ibuprofen/paracetamol combination over ibuprofen and paracetamol in monotherapy are reported in the literature [104,106,107,108,109].

From our point of view, these studies present some limitations that may have contributed to unsuccessful results. For instance, Hung et al. [109] reported no difference in the analgesic efficacy and side effects of the ibuprofen/paracetamol combination over both drugs administered in monotherapy. However, in this study, the drugs were not administered simultaneously. Also, Ianiro et al. [108], Wells. et al. [107], and Kellstein D, Leyva R. [106] reported unsuccessful results; however, in their studies, only one monotherapy group was included as a comparator. In our view, the inclusion of both paracetamol and ibuprofen monotherapy in parallel groups seems to be relevant since, in some works, the authors reported an analgesic improvement of the combination only over one of the monotherapy groups. For instance, Dahl et al. [103], Doherty M. et al. [105], and Thybo at al., 2019 [104] reported a superior analgesic effect in the combination group when compared to paracetamol in monotherapy but not when compared to ibuprofen alone.

Attending to such, we propose the need to design specific clinical trials to quantify the real impact of new NSAID-based FDCs. Clinical trials where the main goal is to assess the efficacy and safety of an already formulated FDC (instead of using drugs in separated formulations), and where parallel arms of both drugs alone as comparators are included, will be needed. Nevertheless, since the combination of ibuprofen/paracetamol is already on the market, it is not the goal of our work to explore the clinical evidence of this combination. In the future, we believe that this point will deserve a deep review and an extensive analysis.

### 3.2. Metamizole-Based Parenteral Fixed-Dose Combinations

As reported previously, like paracetamol, metamizole is a very popular analgesic. However, it was banned in the USA, the UK, Canada, Australia, Japan, Sweden, Denmark, and India since it is related to the occurrence of severe cases of neutropenia and agranulocytosis [51,52]. However, in some European countries, Latin America, Israel, and Russia, metamizole is yet the most-consumed non-opioid analgesic, being used as a first-line analgesic and available as an over-the-counter medication. Germany is one of the most relevant European countries where metamizole is largely consumed [51,52,111]. In 2016, the prescription volumes of metamizole Increased more than 8-fold, reaching a total of 204 million defined daily doses (DDD), which equals 2.9 DDD per person per year [51]. Interestingly, despite the high consumption of metamizole in Germany, all metamizole-containing combinations were withdrawn and banned from the German market in 1987 [53].

Since the number of parenteral-metamizole-based FDCs in Europe is very scare and inexistent in the US, a larger search was performed to include the worldwide market. For this, all metamizole-based combinations found on Vademecum.es, Drugs.com, PubMed, Web of Science, Google Scholar, and Clinicaltrials.com are presented below (Table 2). Some parenteral FDCs of metamizole with antispasmodics, muscular relaxants, antihistamines, Vitamin B_12_ and corticosteroids, penicillin, and expectorants are available on the market. In contrast, no parenteral FDCs composed of NSAIDs with metamizole were found.

Recently, a randomized clinical trial (RCT) assessing the potential benefit of the combination of metamizole and ibuprofen after third lower molar extraction was concluded (NCT02686021) [112] (below Section 3.3.1). In the future, more clinical trials assessing the efficacy of combinations with NSAIDs and metamizole should be conducted to produce solid clinical evidence concerning such combinations.

**Table 2:** Fixed-dose combinations with paracetamol- and metamizole-based medicines available on the market.

Combination	Strengths	Route of Administration	Indications	Brands ^a^	Countries with Marketing Authorization ^b^
Paracetamol + Ibuprofen	1000 mg + 300 mg/ 100 mL	IV	Short-term symptomatic treatment of moderate acute pain in adults when intravenous administration is considered clinically necessary and/or when other routes of administration are not possible [113].	Combofusiv^®^ Comboval^®^ Combogesic^®^	AT, CZ, DE, EE, IE, HR, HU, LT, MT, NL, PT, SI, SE, and UK
Metamizole + Scopolamine (or Butylscopolamine)	2500 mg + 20 mg/5 mL	IM/IV	Post-surgical pain, post-trauma pain, or colicky pain [114].	Buscapina^®^ compositum	AR, BR, CL, ES, and MX
Metamizole + Pitofenone	2500 mg + 10 mg/5 mL	IM/IV	Treatment of painful conditions in the digestive tract and in the bile and urinary tract [115].	Litalgin^®^	FI
Metamizole + Pitofenone + Fenpiverinium	2500 mg + 10 mg + 0.1 mg/5 mL	IM/IV	Treatment of painful conditions in the digestive tract and in the bile and urinary tract and dysmenorrhea [116].	Analgin^®^ Spasmalgon^®^	CZ, LV, and PL
Metamizole + Adiphenine + Promethazine	750 mg + 25 mg + 25 mg/2 mL	IM	General painful conditions [117].	Dorilen^®^	BR
Metamizole + Hydroxocobalamin + Dexamethasone	500 mg + 5 mg + 2 mg/1 mL	IM	Acute joint inflammation processes such as arthritis, periarthritis, bursitis, gout, ankylosing, and spondylitis; in degenerative processes that go along with pain, such as arthrosis and intervertebral disc disorders; in neuralgia; and in back and neck pain [118].	Dexalgen^®^	BR
Metamizole + Pargeverine	2000 mg + 5 mg/4 mL	IV	Treatment of all acute pain accompanied by muscle spasms in any portion of the digestive, hepatobiliary, or urinary tracts or the female genitals [119].	Viadil^®^ Compuesto	CL
Metamizole + Ampicillin + Guaifenesin + Lidocaine + Chlorphenamine	500 mg + 500 mg + 100 mg + 30 mg + 4 mg/3 mL	IM	Unknown ^c^	Ampigrin^®^	MX
Metamizole + Procaine Penicillin G	400,000 U.I. + 500 mg/5 mL	IM	Unknown ^c^	Respicil^®^	MX
Metamizole + Chlorphenamine	Unknown ^c^	Unknown ^c^	Unknown ^c^	Singril^®^ iny	MX

Abbreviations: IM—intramuscular, IV—intravenous, IM/IV—intramuscular and intravenous. ^a^ Here are presented only the main brands found during the research. However, in many cases, there are available on the market other products with other brand names. ^b^ The countries are listed in two-letter country codes. ^c^ Information about these products is vary scare and only available on commercial websites. The summary of product characteristics of these products was not found.

### 3.3. Other NSAID-Based Combinations

Since the unique ready-to-use parenteral NSAID-based FDC available on the market is the paracetamol/ibuprofen developed by AFT Pharmaceuticals, we performed a literature search to study the clinical evidence of the efficacy and safety of combining other NSAIDs with paracetamol or metamizole. This search was performed using PubMed, Google Scholar, and Web of Knowledge, and all RCTs published since were considered. The literature search included keywords such as NSAID, paracetamol, propacetamol, metamizole, dipyrone, combination, fixed-dose, ready-to-use, and NSAID, and the exact search string used was “(NSAID OR acetam* OR paracet* OR proparacet*) AND (fixed* OR FDC OR ready* OR combin* OR multimodal OR analg*) AND (surgery OR oper* OR acute OR post* OR pain) AND adults” and resulted in the discovery of more than 8000 references. The titles and abstracts were analyzed for relevance and included to be reviewed according to inclusion and exclusion conditions.

The published papers were reviewed if they reported results about RCTs performed in adult humans aiming to compare the efficacy and/or safety profiles of combinations of NSAIDs with paracetamol or metamizole. Studies with propacetamol (PPCM) were also included due to its well-known similarity to paracetamol. Due to the lack of RCTs for parenteral FDCs, all types of pain, all systemic routes of administration (enteral and parenteral), and all dose regimens were considered. For the same reason, studies where the combined drugs were not administered simultaneously or by the same route were also included. On the other hand, studies where the combination was locally administered via spinal and intra-articular routes were excluded since the action of the drugs was local and not systemic. Also, studies with opioid analgesics as comparators, studies with pediatric enrolment, and other trials where the aim was not to treat painful conditions (e.g., ductus arteriosus) were also excluded.

Apart from that, all RCTs related to ibuprofen/paracetamol combinations were also excluded. These studies were excluded since this combination is already on the market and the aim of this evaluation is to find clinical evidence related to the efficacy and safety of NSAID-based combinations that are not available yet as FDCs. In total, 17 RCTs [95,96,97,98,99,100,101,102,103,104,105,106,107,108,109,120,121] where the ibuprofen/paracetamol combination was used for different painful conditions were excluded from our review. Applying the inclusion and exclusion criteria described above, 19 references were considered in this review. These works are presented in further detail in Table 3 and report the clinical assessment of paracetamol/propacetamol or metamizole combinations with diclofenac, ibuprofen, ketoprofen, ketorolac, and piroxicam.

Table 3 is sorted primarily by metamizole or by paracetamol used in the combination (alphabetical order), secondly by NSAID used in the combination (alphabetical order), and thirdly by year of publication of the paper (chronological order). The table includes 1 RCT assessing the efficacy and safety of combining ibuprofen with metamizole, 10 RCTs combining diclofenac with paracetamol (or propacetamol), 5 RCTs combining ketoprofen with paracetamol (or propacetamol), 2 RCTs combining ketorolac with paracetamol (or propacetamol), and 1 combining piroxicam with paracetamol. The vast majority of the RCTs presented in Table 3 were randomized, parallel, double-blind, and controlled clinical trials. However, there are some exceptions. Montgomery et al. [122] assessed the analgesic efficacy of paracetamol alone and in combination with diclofenac in an open-label study. Also, Romundstad et al. [123] designed a crossover trial instead of a parallel one. There, according to the authors, the patients were randomized in blocks of four in a complete crossover manner according to a balanced, reduced Latin square design. Recently, Msolli et al. [124] opted to explore the possible benefits of combining piroxicam with paracetamol in a single-blind study instead of using the typical double-blind approach. In this case, the randomization was performed by a blinded study investigator.

Concerning the pain models, all except one study used real clinical painful conditions to assess the analgesic combinations. Romundstad et al. [123] used an artificial approach to generate a painful stimulus in healthy volunteers. The measurement of pain intensity with a visual analog scale (VAS) and the measurement of opioid drug consumption during a given period were the most common primary outcomes applied in the studies to quantify the impact of the interventions.

Beyond the acquisition of clinical evidence, the development of new FDCs brings new challenges to overcome, mainly parenteral-products-related ones. In this way, beyond the expectable challenges related to the development of parenteral products, the formulation of a parenteral FDC could be even more complex [22,125]. During the development of new parenteral formulations, critical quality attributes, such as the pH, tonicity, fill volume, shelf-life, and packaging conditions, must be considered and deeply investigated [22]. In the case of a parenteral FDC, the definition of such attributes could be more complex due to the necessity of assessing and ensuring the stability of the combined drugs and the chemical compatibility between them [22,125,126,127]. After development, additional regulatory requirements must be considered during the application for the marketing authorization of new FDCs [126]. According to the European Guideline on the clinical development of fixed-combination medicinal products (EMA/CVMP/83804/2005), the basic scientific requirements for any fixed-combination medicinal product are as follows [128,129]:

1. Justification and rationale for the combination.
2. Demonstration of the contribution of all active substances to the desired therapeutic effect.
3. The relevance of the evidence presented to the fixed-combination medicinal product.

Also, additional drug–drug interaction studies may be required to assess the pharmacodynamics and pharmacokinetics of the FDC [129].

In contrast to Europe, in the US, the FDA provides two guidance documents for drug combinations, but none of these was specifically issued to provide concrete instructions for the development of new FDCs [130]. One document is specifically related to FDCs, titled “Fixed Dose Combinations, Co-Packaged Drug Products, and Single-Entity Versions of Previously Approved Antiretrovirals for the Treatment of HIV” (FDA-2013-S-0610) [131]; there is another related to combinations therapy, titled “Codevelopment of Two or More New Investigational Drugs for Use in Combination” (FDA-2010-D-0616) [132]. Since the FDA does not have specific instructions, its decisions during the assessment of new FDCs are flexible, if well justified [130].

**Table 3:** Published randomized clinical trials involving NSAIDs combinations with paracetamol or propacetamol.

Author, Year	Trial Design (N)	Pain Model	Study Objectives	Treatment Details (Drug; Dosage; Regimen; Route; Frequency; Duration)	Primary Outcomes	Conclusions
Metamizole Combinations						
Ibuprofen + Metamizole						
Schneider et al., 2022 [112]	Randomized, crossover, double-blind, and controlled trial (35)	Lower third molar extraction	To compare the combination of ibuprofen/metamizole with either drug alone in relieving postoperative pain.	1. MTZ 1000 mg 2. IBP 400 mg 3. MTZ 1000 mg + IBP Each patient received three applications and was assessed for 18 h.	Mean pain score	Efficacy: Combined use enables superior pain control compared to ibuprofen alone and tends to be superior to metamizole alone. Safety: Not addressed by the authors.
Paracetamol (or Propacetamol) Combinations						
Diclofenac + Paracetamol (or Propacetamol)						
Montgomery et al., 1996 [122]	Randomized, parallel, open-label, and controlled trial (60)	Elective abdominal gynecological surgery	Assess the analgesic efficacy of paracetamol alone and in combination with diclofenac.	1. DCF 100 mg 2. APAP 1500 mg 3. DCF 100 mg + APAP 1500 mg Single rectal dose was given before the surgery with 24 h of observation.	Opioid consumption after the operative procedure	Efficacy: Combination reduced the amount of morphine consumed. Safety: No difference in the incidence of side effects between the groups.
Breivik et al., 1999 [133]	Randomized, parallel, double-blind, and controlled trial (120)	Surgical removal of third molars	Assess the analgesic effect of combining diclofenac with paracetamol and with codeine.	1. DCF 100 mg 2. APAP 1000 mg 3. DCF 100 mg + APAP 1000 mg 4. DCF 100 mg + APAP 1000 mg + CDN 60 mg 5. APAP 100 mg + CDN 60 mg Single oral dose was given after the surgery with 8 h of observation.	Pain intensity (VAS score)	Efficacy: Combination of drugs is superior to diclofenac or paracetamol alone. Safety: No difference in the incidence of side effects between the groups.
Beck et al., 2000 [134]	Randomized, parallel, double-blind, and controlled trial (70)	Hysterectomy	Assess the pharmacokinetics of rectal paracetamol in women and compare their analgesic efficacy with a diclofenac combination.	1. APAP 20 mg/kg (small-dose) 2. APAP 40 mg/kg (large-dose) 3. DCF 100 mg + APAP 20 mg/kg Single rectal dose was given before the surgery within 24 h of observation.	Opioid consumption Pain intensity (VAS score).	Efficacy: Only lower VAS scores after APAP + DCF at 4 h. Safety: Not addressed by the authors.
Siddik et al., 2001 [135]	Randomized, parallel, double-blind, and controlled trial (80)	Cesarean	Assess the postoperative analgesic effects of propacetamol in combination with diclofenac.	1. Placebo 2. DCF 100 mg 3. PPCM 2 g 4. DCF 100 mg + PPCM 2 g Propacetamol intravenously q.i.d and diclofenac rectally t.i.d over 24 h following surgery.	Opioid consumption Pain intensity (VAS at rest and on coughing) Patient satisfaction	Efficacy: No statistical difference between the groups. Safety: No difference in the incidence of side effects between the groups.
Man et al., 2004 [136]	Randomized, parallel, double-blind, and controlled trial (50)	Painful soft-tissue injuries	Asses the efficacy and safety of oral paracetamol compared with NSAIDs or combination therapy.	1. DCF 25 mg 2. APAP 1000 mg 3. DCF 25 mg + APAP 1000 mg 4. IND 25 mg Oral administration of diclofenac t.i.d, paracetamol q.i.d, and indomethacin t.i.d with observation over 120 min (stage 1) and 3 days (stage 2).	Pain intensity (VAS score) at rest and with movement	Efficacy: No statistical difference between the groups. Safety: No difference in the incidence of side effects between the groups.
Hiller et al., 2004 [137]	Randomized, parallel, double-blind, and controlled trial (71)	Elective tonsillectomy	Assess the analgesic efficacy between the combination of paracetamol with diclofenac and either drug alone.	1. DCF 75 mg 2. PPCM 2000 mg 3. DCF 75 mg + PPCM 2000 mg Single IV dose was administered after anesthetic induction and postoperatively; propacetamol was administered twice and diclofenac once.	Pain intensity (VRS and VAS scores) at rest and on swallowing	Efficacy: No statistical difference between the groups. Safety: No difference in the incidence of side effects between the groups.
Woo et al., 2005 [138]	Randomized, parallel, double-blind, and controlled trial (300)	Musculoskeletal Injury	Assess the efficacy safety of oral paracetamol compared with oral nonsteroidal anti-inflammatory drugs or combination therapy.	1. DCF 25 mg 2. APAP 1000 mg 3. DCF 25 mg + APAP 1000 mg 4. IND 25 mg Stage 1—single oral dose with 2 h of observation. Stage 2—outside the hospital, the same therapy with 3 days of observation (paracetamol q.i.d and diclofenac or indomethacin t.i.d)	Pain intensity (VAS) at rest and with limb movement	Efficacy: The analgesic benefits of oral combination were small and of doubtful clinical significance. Safety: No difference in the incidence of side effects between the groups.
Legeby et al., 2005 [139]	Randomized, parallel, double-blind, and controlled trial (50)	Mastectomy with immediate breast reconstruction	Assess the analgesic efficacy of diclofenac in combination with paracetamol and opioids.	1. APAP 1000 mg 2. DCF 50 mg + APAP 1000 mg Diclofenac was administered rectally t.i.d and paracetamol was administered orally t.i.d with 64 h of observation.	Pain intensity (VAS score)	Efficacy: The combination reduced opioid consumption and improved pain relief during the first 20 h. Safety: Post-operative bleeding was significantly higher with diclofenac than with a placebo (*p* < 0.01).
Munishankar et al., 2008 [140]	Randomized, parallel, double-blind, and controlled trial (78)	Elective cesarean section	Assess the efficacy of the combination of diclofenac and paracetamol used for pain relief after major surgery.	1. DCF 100 mg 2. APAP 1000 mg 3. DCF 100 mg + APAP 1000 mg Study drugs were given as a suppository at the end of surgery and then orally for 24 h. Paracetamol q.i.d and diclofenac t.i.d with 24 h of observation.	Opioid consumption	Efficacy: Patients given a combination of diclofenac and paracetamol used 38% less morphine compared to patients given paracetamol. Safety: Not addressed by the authors.
Ridderikhof et al., 2018 [141]	Randomized, multicenter, parallel, double-blind, and controlled trial (547)	Acute musculoskeletal trauma	Assess the efficacy of paracetamol and diclofenac alone or in combination.	1. DCF 50 mg 2. APAP 1000 mg 3. DCF 50 mg + APAP 1000 mg Diclofenac and paracetamol were administered orally t.i.d and q.i.d, respectively, with 3 days of observation.	Pain intensity (NRS pain score) at rest and with movement	Efficacy: No statistical difference between the groups. Safety: No difference in the incidence of side effects between the groups.
Ketoprofen + Paracetamol (or Propacetamol)						
Fletcher et al., 1997 [142]	Randomized, parallel, double-blind, and controlled trial (60)	Disc surgery	Assess the effect of combining propacetamol with ketoprofen.	1. KTPF 50 mg 2. PPCM 2000 mg 3. KTPF 50 mg + PPCM 2000 mg 4. Placebo All drugs were given q.i.d and intravenously for 2 days after the surgery. The observation occurred during the same period.	Pain intensity (VAS score)	Efficacy: The combination reduced pain scores both at rest and on movement. Safety: No difference in the incidence of side effects between the groups.
Aubrun et al., 2000 [143]	Randomized, parallel, double-blind, and controlled trial (50)	Spinal fusion surgery	Assess the efficacy of ketoprofen in patients receiving propacetamol.	1. PPCM 2000 mg 2. KTPF 100 mg + PPCM 2000 mg Ketoprofen and propacetamol were administered intravenously t.i.d and q.i.d, respectively, over 24 h following surgery. The observation occurred during the same period.	Pain intensity (VAS score)	Efficacy: The combination reduced morphine requirements and improved postoperative analgesia. Safety: No difference in the incidence of side effects between the groups.
Fourcade et al., 2005 [144]	Randomized, parallel, double-blind, and controlled trial (97)	Thyroidectomy	Compare the efficacy of propacetamol and ketoprofen, alone or in combination.	1. KTPF 100 mg 2. PPCM 2000 mg 3. KTPF 100 mg + PPCM 2000 mg Propacetamol and Ketoprofen were administered intravenously 30 min before the end of surgery and 6 and 12 h after the surgery. The observation occurred during the same period.	Pain intensity (VAS score)	Efficacy: No statistical difference between the groups. Safety: Not addressed by the authors.
Akural et al., 2009 [145]	Randomized, parallel, double-blind, and controlled trial (76)	Postoperative dental pain	Assess the efficacy of combining paracetamol with ketoprofen.	1. KTPF 100 mg 2. APAP 1000 mg 3. KTPF 100 mg + APAP 1000 mg 4. Placebo Ketoprofen and paracetamol were administered orally in a single dose. The observation was performed every 15 min for 10 h.	Pain intensity difference (PID), sum of PID (SPID), and NRS score at rest and on dry swallowing	Efficacy: The combination provided a significantly more rapid onset of analgesia than either drug alone. Safety: No difference in the incidence of side effects between the groups.
Salonen et al., 2009 [146]	Randomized, parallel, double-blind, and controlled trial (116)	Tonsillectomy	Evaluate the efficacy of co-administration of intravenous paracetamol with ketoprofen.	1. KTPF 1 mg/kg 2. KTPF 1 mg/kg + APAP 1000 mg 3. KTPF 1 mg/kg + APAP 2000 mg Both ketoprofen and paracetamol were administered intravenously in a single dose after the surgery.	The proportion of patients requiring rescue analgesia	Efficacy: In the combination groups, the number of opioid doses was reduced. Safety: No difference in the incidence of side effects between the groups.
Ketorolac + Paracetamol (or Propacetamol)						
Romundstad et al., 2006 [123]	Randomized, crossover, double-blind, and controlled trial (16)	Pressure algometry	Evaluate the efficacy of propacetamol 2 g and ketorolac 30 mg, individually and in combination.	1. KTLC 30 mg 2. PPCM 2000 mg 3. KTLC 30 mg + PPCM 2000 mg 4. Placebo The crossover study had a Latin square design. The drugs were administered intravenously in a single dose and the observation was performed for 165 min.	Pressure pain tolerance threshold (PPTT)	Efficacy: Combining paracetamol with ketorolac increased the PPTT. Safety: No difference in the incidence of side effects between the groups.
Iorno et al., 2013 [147]	Randomized, parallel, patient-blinded, and controlled trial (60)	Voluntary ambulatory abortion	Assess the efficacy and safety of oral paracetamol with IV ketorolac.	1. KTLC 30 mg 2. KTLC 30 mg + APAP 1000 mg Ketorolac was administered intravenously o.d and paracetamol was administered orally t.i.d. The patients were observed until the following morning.	Pain intensity (NRS score)	Efficacy: The studied drugs were effective and well tolerated in the control of postoperative pain. Safety: No difference in the incidence of side effects between the groups.
Piroxicam + Paracetamol (or Propacetamol)						
Msolli et al., 2021 [124]	Randomized, parallel, single-blinded, and controlled trial (1632)	Traumatic injury	Explore the possible benefits of combining piroxicam with paracetamol.	1. APAP 1000 mg 2. PRX 20 mg 3. PRX 20 mg + APAP 1000 mg Paracetamol and piroxicam were administered orally t.i.d and b.i.d, respectively. Each patient was re-evaluated on the 3rd and 7th days.	Need for additional oral analgesics	Efficacy: The combination did not increase the analgesic effect compared to paracetamol alone. Safety: The occurrence of adverse events was significantly more frequent in the PRX alone and combination groups.

Abbreviations: APAP—paracetamol, CDN—codeine, DCF—diclofenac, FDC—fixed-dose combination, IBP—ibuprofen, IND—indomethacin, IM—intramuscular, IV—intravenous, IM/IV—intramuscular and intravenous, KTLC—ketorolac, KTPF—ketoprofen, MTZ—metamizole, NRS—numeric rating scale, NSAID—non-steroidal anti-inflammatory drug, o.d—once a day, q.i.d—four times a day, t.i.d—three times a day, PID—pain intensity difference, PPCM—propacetamol, PPTT—pressure pain tolerance threshold, PRX—piroxicam, SPID—sum of pain intensity difference, VAS—visual analog scale, VRS—verbal rating scale.

All studies presented in Table 3 reported the same analgesic dosages and routes of administration commonly used and authorized in monotherapy. In other words, none of these studies considered the use of sub-therapeutic doses to assess the synergic effect by combining analgesics nor suggested alternative routes using new formulations. Beck et al. [134] published results where two different doses of paracetamol were introduced in two parallel arms of the trial to compare their efficacy with a diclofenac combination. In this work, the efficacy and pharmacokinetics of lower (20 mg/kg) and higher (40 mg/kg) doses of paracetamol were compared to the combination of diclofenac (100 mg) with paracetamol (20 mg/kg) [134].

Although the reviewed RCTs aimed to assess efficacy and safety profiles by combining NSAIDs with metamizole or paracetamol (or propacetamol), the combined drugs were not always administered by the same route or at the same time. These studies cannot directly assess the potential value for the development of new analgesic FDCs; yet, they could serve as proof-of-concept works by displaying their therapeutic utility. At this point, it is important to underscore that these studies were not designed to assess the efficacy or the safety profiles of FDCs but to assess the benefits of analgesics use in combination for the treatment of painful conditions. In two studies, the route of administration of the combined drugs was not the same. Siddik et al. [135] designed a trial where propacetamol was administered intravenously, while diclofenac was rectally applied. Also, in the study by Iorno et al. [147], ketorolac was administered intravenously and paracetamol was administered orally. Concerning the synchronicity related to the administration of the drugs, although the reviewed RCTs aimed to assess efficacy by combining analgesic drugs, some reviewed works reported an asynchronous administration of the drugs rather than a simultaneous administration, as achieved with FDC formulations. Siddik et al. [135] reported a trial protocol where 100 mg of diclofenac was rectally administered every 8 h and 2 g of propacetamol was administered intravenously every 6 h. Also, Man et al. [136], Woo et al. [138], and Ridderikhof et al. [141] reported the combination of diclofenac with paracetamol in tablets based on t.i.d. and q.i.d. regimens, respectively. Yet, Munishankar et al. [140] administered diclofenac t.i.d. and paracetamol q.i.d.; however, in this case, they used 50 mg diclofenac and 1000 mg paracetamol rectally at the end of the surgery and then orally during the patients’ recovery [140]. Also, combining ketoprofen and propacetamol, Aubrun et al. [143] administered the NSAID every 8 h and propacetamol every 6 h. However, in this case, the drugs were both administered intravenously. Concerning the clinical trial performed by Iorno et al. [147], ketorolac was applied orally and intravenously based on an o.d. regimen and paracetamol every 8 h. Very recently, in the RCT published by Msolli et al. [124], the authors conducted a trial combining piroxicam b.i.d. and paracetamol every 8 h [124].

In some trials, although the combined drugs were both administered simultaneously and through the same route of administration, none of the authors claimed the intention to develop or at least assess the possibility of proposing the development of FDCs. Despite all this, and focusing attention on parenteral FDCs, some studies presented in Table 3 could be useful to assess the clinical impact of the development of new parenteral FDCs. Concerning the trials about combinations with diclofenac, Montgomery et al. [122] and Beck et al. [134] reported the simultaneous administration of the combination as a single rectal dose given before the surgery procedure. Breivik et al. [133] also reported the administration of a single rectal dose, but after the surgery procedure. In the same way, in the trial published by Legeby et al. [139], diclofenac and paracetamol were administered simultaneously but rectally and orally, respectively. Yet, in the work published by Hiller et al. [137], the first dose of combined drugs was administered simultaneously via IV right before the surgery procedure but, post-operatively, the administration of the combination was not always performed simultaneously. In the case of ketoprofen combinations, only in the work published by Aubrun et al. [143] was the administration of the combined drugs not performed simultaneously.

Concerning the ketoprofen RCTs presented in Table 3, the most reported route of administration was IV. Indeed, only the study performed by Akural et al. [145] reported an oral administration of the referred combinations. Also, Salonen et al. [146] administered ketoprofen with propacetamol intravenously in a simultaneous manner and in a single-dose regimen. Fletcher et al. [142] reported the simultaneous IV administration of the combination every 6 h for 2 days after surgery. In the same way, in the study performed by Fourcade et al. [146], the patients received the combined drugs but only for 12 h.

In the case of ketorolac combinations, in the literature were found two RCTs where ketorolac was combined with paracetamol or propacetamol (Table 3). In a trial performed by Romundstad et al. [123], both ketorolac and propacetamol were used intravenously in a single dose. In the case of Iorno et al. [147], ketorolac was administered intravenously only at the end of the surgical intervention, while paracetamol was applied 15 min before surgery, on discharge from the hospital, and in the morning of the day after surgery.

A study where piroxicam was combined with paracetamol was published recently to explore the possible benefits of combining the two drugs. In this study, both drugs were administered orally but not at the same time [124].

Regarding the efficacy and safety results and considering the works presented in Table 3, some but not all authors were able to demonstrate superior analgesia or superior clinical outcomes using combinations of NSAIDs with paracetamol or metamizole for the treatment of acute painful conditions.

#### 3.3.1. Metamizole-Based Combinations with Ibuprofen

During our literature search, only one work assessing the efficacy of combining NSAIDs with metamizole was found. The full text of this work is not available; however, according to the information available on the clinicaltrails.gov website, the route of administration of the combined drugs is unknown and it does not report the use of FDC but only the use of metamizole and ibuprofen in combination.

According to the abstract of the work published by Schneider et al. [112], the combined treatment of ibuprofen/metamizole showed lower mean pain scores over 12 h than ibuprofen (2.4 ± 1.3 vs. 3.8 ± 1.6; *p* = 0.005) and showed lower mean pain scores over 6 h than ibuprofen (2.0 ± 1.2 vs. 3.1 ± 1.6; *p* = 0.022) and metamizole (2.0 ± 1.2 vs. 3.3 ± 1.7; *p* = 0.015) alone. Also, the consumption of rescue medication was lowest in the combination-group (25% vs. 46%-metamizole and 50%-ibuprofen) (Table 3).

In the future, more clinical evidence of this kind is required. We believe that the combination of metamizole with NSAIDs should be deeply studied in the future since metamizole is banned in some countries, but it is extensively consumed worldwide [33].

#### 3.3.2. Paracetamol-Based Combinations with Diclofenac

According to our literature review, the combination most reported in published works is the combination of diclofenac with paracetamol (or propacetamol).

Montgomery et al. [122] designed an RCT (*n* = 60) aiming to compare the analgesic efficacy of the combination of diclofenac/paracetamol with the efficacy of both drugs alone. The outcome used in this trial was the morphine intake by patients. The authors concluded that the use of the combination significantly reduced the amount of morphine consumed when compared with the paracetamol group (*p* < 0.01). Compared with the diclofenac group, the amount of morphine consumed by the combination group was also reduced, but not significantly. Breivik et al. [133] (*n* = 120) investigated the enhanced analgesic effect by combining diclofenac with paracetamol after the removal of third molars in adult patients. The primary efficacy measure was pain intensity and it was concluded that the concomitant oral administration of diclofenac and paracetamol is superior to diclofenac or paracetamol alone (*p* < 0.05). The study performed by Beck et al. [134] (*n* = 70) was somewhat different from the others since its main objective was not to assess the efficacy of a diclofenac/paracetamol combination for the treatment of painful conditions related to gynecological surgery but mainly to study the pharmacokinetics of two doses of paracetamol. In this trial, the authors mainly studied the relationship between plasma concentrations and the analgesic effect of paracetamol. This study did not have a comparator group with diclofenac alone but, in any case, the VAS scores achieved by the combination group were significantly lower (*p* < 0.05) compared with the paracetamol group. Siddik et al. [135] (*n* = 80) evaluated analgesic capability by combining diclofenac with propacetamol in women after cesarean delivery. Based on the VAS score values, some points of observation resulted in favorable and significant results that were observed in the combination group. Despite these results, the authors concluded that this study was unable to show a significant morphine-sparing effect using a combination of diclofenac with propacetamol. Concerning safety, the authors did not find any difference regarding the incidence of adverse events between the assessed groups. Man et al. [136] (*n* = 50) also designed a trial to investigate efficacy and safety profiles by combining diclofenac with paracetamol for the treatment of pain induced by painful soft-tissue injuries treated in an emergency department. In this study, in addition to the groups receiving the combined drugs and the respective drugs alone, the authors inserted another trial arm where patients received indomethacin alone. Based on the results, the authors reported no statistical difference among all the arms concerning both drug efficacy and the incidence of adverse events. Accordingly, Woo et al. [138] designed an identical study but with increased enrolment (*n* = 300). Also, in this study, a small and doubtful clinical utility of the diclofenac with paracetamol combination was reported. Hiller et al. [137] (*n* = 71) also tested the analgesic capability of diclofenac and propacetamol in combination for the treatment of post-operative pain related to elective tonsillectomy in adults. The incidence of pain at rest was significantly lower in the propacetamol group than in the diclofenac group (*p* < 0.05), but not this was not significant compared to the combination group using both drugs alone. In conclusion, the combination of diclofenac offers only a minor advantage concerning postoperative analgesia or the incidence of side effects, without clear clinical advantages in adult tonsillectomy patients. Legeby et al. [139] (*n* = 50) performed an RCT to evaluate the combination of diclofenac with paracetamol. This clinical trial had two arms: one testing the analgesic activity of a combination of diclofenac with paracetamol and another using paracetamol alone. In this work, a trial arm with diclofenac alone was not included since the authors treated the inclusion of diclofenac in the combination group as an added agent to be compared with the control group (paracetamol alone). The authors reported lower VAS scores in the combination group when compared to the control group (*p* < 0.05) in the first 20 h of observation. The authors concluded that adding diclofenac to paracetamol reduced opioid consumption and improved pain relief during the first 20 h at rest but was not effective during patients’ mobilization. Munishankar et al. [140] (*n* = 78) tested the efficacy of combining diclofenac with paracetamol for the pain management of women subjected to elective cesarean section. As the main endpoint, the investigators quantified the consumption of morphine for the first 24 h after the medical procedure. The authors concluded that the consumption of morphine was reduced by 38% in the combination group when compared to the group using paracetamol alone, but the same was not observed when the combination group was compared to the diclofenac group. Concerning the incidence of adverse events, the results suggest that the combination may be associated with more side effects; however, larger studies are required. In a larger study, Ridderikhof et al. [141] (*n* = 547) assessed the efficacy and safety of a diclofenac/paracetamol combination with an RCT for the treatment of acute musculoskeletal traumatic pain. This study aimed to assess the non-inferiority of paracetamol over nonsteroidal anti-inflammatory drugs alone or in combination for pain treatment. The authors concluded that analgesic treatment with paracetamol was not inferior to the analgesia induced by diclofenac or by a combination of diclofenac/paracetamol. Indeed, the intention-to-treat analysis revealed a mean NRS reduction at rest of −1.23 (95% confidence interval [CI] −1.50 to −0.95) and of −1.72 (95% CI −2.01 to −1.44) with movement, both for paracetamol at 90 min compared with the baseline. Pairwise comparison at rest with diclofenac showed differences of −0.027 (97.5% CI −0.45 to 0.39) and −0.052 (97.5% CI −0.46 to 0.36) for combination treatment. With movement, these numbers were −0.20 (97.5% CI −0.64 to 0.23) and −0.39 (97.5% CI −0.80 to 0.018), respectively.

Concerning efficacy, from the 10 studies presented in Table 3, only the study published by Breivik et al. [133] was able to report that the concomitant oral administration of diclofenac and paracetamol is superior to diclofenac or paracetamol alone. Montgomery et al. [122] reported that the combination significantly reduced the amount of morphine consumed by the combination group when compared to the paracetamol group but not when compared to the diclofenac group. Interestingly, Munishankar et al. [140] also reported that the combination was able to demonstrate a superior opioid-sparing effect when compared with the paracetamol group but not with the diclofenac group. In the rest of the studies, the authors were not able to demonstrate any clear advantages of using diclofenac combinations.

Regarding safety, in general, all the studies that published results reported no statistical significance between the combination and monotherapy groups.

#### 3.3.3. Paracetamol-Based Combinations with Ketoprofen

As with diclofenac combinations, some authors assessed the possibility to improve analgesia outcomes by combining ketoprofen with paracetamol or propacetamol.

The clinical study performed by Fletcher et al. [142] (*n* = 60) aimed to evaluate the effect of a combination of ketoprofen (Profenid^®^) with propacetamol (Prodafalgan^®^) after surgery for patients with a herniated disc of the lumbar spine. The authors began with the principle that a combination of analgesic drugs may enhance analgesia and reduce side effects after surgery. The VAS scores obtained from the enrolled patients were used as the primary endpoint of the study and were lower in the combination group than in groups using propacetamol, ketoprofen, and placebo at rest (*p* < 0.05) and on movement (*p* < 0.01). The authors concluded that the combination of propacetamol and ketoprofen effectively reduced pain scores both at rest and on movement but was not able to reduce morphine consumption or the incidence of side effects. Aubrun et al. [143] (*n* = 50) also assessed the analgesic effects of ketoprofen in patients undergoing spinal fusion surgery and receiving propacetamol. As was done by Legeby et al. [139] for the diclofenac combination, also in this case, the NSAID was given as an addition to the propacetamol treatment. Pain intensity was assessed using the VAS scale, and the authors concluded that combining ketoprofen with propacetamol significantly reduced morphine consumption (25 ± 17 vs. 38 ± 20 mg, *p* = 0.04) and VAS scores (*p* = 0.002). Indeed, the total post-operative morphine consumption was significantly reduced (−33%) in the combination group. Fourcade et al. [144] (*n* = 97) compared the efficacy of ketoprofen and propacetamol alone and in combination in adult patients after being subjected to thyroidectomy. The authors explained that although the combination of NSAIDs with NOAs is widely used in clinical contexts, this practice has been poorly assessed. The results achieved in this study revealed that although pain scores were significantly higher with propacetamol compared with ketoprofen and the combination at 2 h after surgery (35 ± 3.7 and 21 ± 2.6, respectively; *p* < 0.01), the concomitant use of propacetamol and ketoprofen does not improve analgesia compared with ketoprofen alone. Akural et al. [145] (*n* = 76) tested the use of ketoprofen plus paracetamol in the treatment of dental pain in a single-dose regimen. Again, the authors introduced their work explaining that a combination of analgesic drugs with different pharmacologic properties may be more effective and with fewer adverse events than monotherapy therapies. The authors reported a significantly greater sum of pain intensity difference (SPID) at rest and on swallowing (at 1.5 h) in the combination group and when compared with the paracetamol, ketoprofen, and placebo groups (all *p* < 0.05). Also, the authors reported a significantly smaller mean time to the onset of pain relief at rest and on swallowing in the combination group when compared to the other groups (all *p* < 0.05) and a significantly longer median time to the use of rescue medication in the combination group when compared to the paracetamol (*p* = 0.006) and the placebo (*p* < 0.001) groups. Concerning side effects, the prevalence of trismus, bleeding, and edema was not significantly different between the studied groups. This study concluded that the obtained results support the clinical practice of combining ketoprofen with paracetamol for the management of acute pain. Salomen et al. [146] (*n* = 114) evaluated whether the co-administration of IV paracetamol could enhance the analgesic efficacy of ketoprofen in patients undergoing a tonsillectomy. Although no difference was detected in the proportion of patients receiving oxycodone between the three groups, significantly fewer doses of rescue analgesia were provided in the combination groups when compared with the ketoprofen-alone group (*p* = 0.005). Indeed, 27% less oxycodone was required in the combination group with 1 g of paracetamol (*p* = 0.023) and 38% less was required in the combination group with 2 g of paracetamol (*p* = 0.002). With these results, the authors concluded that combining IV paracetamol with ketoprofen after a tonsillectomy did not reduce the proportion of patients requiring rescue analgesia but reduced the number of opioid doses demanded by patients.

In summary, as can be consulted in Table 3, not all published works were able to demonstrate increased analgesia capabilities by combining ketoprofen with paracetamol or propacetamol. Also, concerning the safety of ketoprofen with an APAP (or PPCM) combination, it seems not to have any significant impact on the occurrence of adverse events.

#### 3.3.4. Paracetamol-Based Combinations with Ketorolac

Ketorolac was the first parenteral NSAID commercialized in the USA in 1990 [148]. Due to their long presence in the market, the very lack of clinical trials assessing the analgesia efficacy of ketorolac combinations with paracetamol or propacetamol is surprising. Concerning the number of studies on the combination of ketorolac with APAP, our results are in accordance with the results obtained in another study published in 2010 by Cliff et al. [149] where the authors performed a qualitative systematic review of the analgesic efficacy of combinations of NSAIDs with paracetamol for the treatment of acute postoperative pain. In this review, only one RCT with ketorolac was reported by the authors.

Romundstad et al. [123] (*n* = 16) assessed analgesic capability when combining ketorolac with propacetamol in healthy patients. For this, the authors applied a pressure stimulus on the base of a fingernail until the pressure pain tolerance threshold was reached. For the total observation period, only in the combination group was observed a significant increase in the pressure pain tolerance threshold (PPTT) when compared with the baseline (*p* < 0.04), and PPTT decreased significantly after the administration of a placebo (*p* < 0.01). Also, the combination and ketorolac alone increased PPTT compared with the placebo (*p* < 0.001) and with propacetamol (*p* < 0.001). Additionally, the combination was significantly superior to ketorolac alone (*p* < 0.04), but not to propacetamol alone since after receiving 2 g of propacetamol, the PPTT did not change significantly compared with either a placebo or the baseline. With these findings, the authors concluded that it was advantageous to combine paracetamol with an NSAID for the relief of acute pain. Also, Iorno et al. [147] (*n* = 60) tested the analgesic efficacy and safety profile of combining ketorolac with paracetamol in enrolled women subjected to voluntary abortion. The authors achieved significant differences in pain levels at T0 (NRS 0.92 and 2.08; *p* < 0.01), T2 (in the morning after surgery; data collected by phone interview), and following the administration of the next dose of paracetamol (1.58 vs. 1.98; *p* = 0.01). Concerning adverse events, only a case of dizziness was reported in the combination group, and no other unexpected adverse events were recorded. With these results, this small study suggests that oral paracetamol t.i.d. in combination with IV ketorolac o.d. is effective and well tolerated in the control of post-operative pain after ambulatory uterine evacuation.

Both studies assessed the efficacy and safety of combining ketorolac with paracetamol or propacetamol/PPCM; however, they are very modest in their confirmation of any conclusive results. Despite all this, both assays present favorable evidence for the use of ketorolac in combination with paracetamol or propacetamol.

#### 3.3.5. Paracetamol-Based Combinations with Piroxicam

Piroxicam is indicated for the symptomatic relief of osteoarthritis, rheumatoid arthritis, and ankylosing spondylitis. However, due to its safety profile, piroxicam should not be a first-line option [67]. The very long half-life of oxicams like piroxicam has been related to increased GI toxicity when compared with other traditional NSAIDs [150]. Probably due to this fact, the number of published works about this NSAID is very limited.

During our literature search, only one study assessing a combination with piroxicam was found. In the RCT performed by Msolli et al. [124] (*n* = 1504), the authors explored the benefits of combining piroxicam with paracetamol in the treatment of post-traumatic pain in patients admitted to an emergency department. The primary outcome was the need for additional oral analgesics. The results indicated that the need for additional oral analgesics was comparable between the paracetamol-NSAID combination group (9.8%) and the paracetamol group (11.4%; *p* = 0.43). Also, the ED readmission rate in the emergency department was similar between the two groups at 5.6 and 5.8%, respectively (*p* = 0.86). In contrast, the need for new analgesics and emergency revisit rates were both more frequent in the piroxicam group, where the frequency of dissatisfaction was higher. Concerning the safety profile, side effects were more frequent in the piroxicam and combination groups. With this, the authors concluded that the combination of piroxicam with paracetamol does not increase the analgesic effect compared to paracetamol alone. Also, they reported that paracetamol alone is superior to piroxicam alone for post-traumatic extremity pain.

## 4. Discussion

The combination of NSAIDs with paracetamol or metamizole is the most common strategy used in multimodal analgesia. Due to the well-known efficacy and safety profiles of paracetamol and NSAIDs, their combination is one of the most recommended multimodal therapeutic strategies in several clinical guidelines for pain management [7,10]. Metamizole is considered an alternative to paracetamol; however, it is banned in some countries due to its doubtful safety profile. Despite all this, metamizole is a good non-opioid analgesic to be considered in the development of new FDC products since several parenteral FDCs with metamizole are already available on the market (Section 3.2).

Despite the large non-opioid analgesics on the market, only one ibuprofen/paracetamol FDC is approved in the European market. This ready-to-use FCD is available in tablets and in a solution for IV administration, but these products have both been introduced to the market recently (Section 3.1). In fact, despite the evident clinical and economic advantages provided by parenteral ready-to-use FDCs, their development and marketing authorization can be very challenging. During development, investigational and regulatory considerations make the product’s development and approval processes more complex (Section 3.3). During development, drug–drug interactions must be studied, ensuring that the drug combination does not change the product’s biopharmaceutical attributes. Also, a formulation compatible with both APIs must be found to ensure the product’s stability [125]. During the application of marketing authorization, a clear advantage of the new FDC when compared with other medicinal products must be demonstrated [90,91,92,125,151]

Considering the availability of parenteral formulations of paracetamol, metamizole, and some NSAIDs in the European and American markets (Section 2.2.1,Section 2.2.2,Section 2.2.3), it is possible, at least conceptually, to develop new parenteral ready-to-use FCDs with these drugs. However, to reach the market, these products must be assessed by well-designed RCTs (Section 3.1). Fortunately, since the efficacy and safety profiles of these drugs are already well established in monotherapy, it is only necessary to demonstrate the safety and efficacy of both drugs in combination. Based on the marketing applications submitted to the EMA for the market introduction of Comboval^®^ and Maxigesic^®^ IV, the non-clinical aspects submitted can be based just on a literature review. In contrast, for the clinical aspects, well-designed RCTs are mandatory to assess the efficacy and safety profiles of the new combination. Also, it is crucial to prove that the combination does not alter the pharmacokinetics of the combined drugs [91,92].

In the literature, there is a lack of RCTs available to study the potential utility of NSAID combinations (Section 3.3). Some authors designed RCTs to assess the efficacy and safety of combining NSAIDs with paracetamol (or propacetamol). However, several of them were unable to demonstrate clear advantages in the use of such combinations (Section 3). In our view, and focusing on the design of the studies, some reviewed works have limitations that may have deeply impacted the reported results. For instance, in some studies, the drugs were not administered simultaneously and/or by the same route. Since these studies were not designed to assess the efficacy nor the safety profiles of FDCs, they could serve as proof-of-concept studies concerning the efficacy and safety of combining NSAIDs with paracetamol or metamizole.

Despite all this, the recent discontinuation of Anjeso (meloxicam IV formulation) raises the question of whether there still exists a space on the market for the introduction of new parenteral products for pain management. In our present investigation, no scientific studies sharing the analgesic’s market by route of administration were found. In the future, studies related to the market share of parenteral NSAIDs and NOAs may be useful to study the market’s viability for the introduction of new parenteral ready-to-use FDCs. Moreover, complex investigational and regulatory considerations and the alleged lack of space on the market may discourage the development of new NSAID-based FDCs.

## 5. Conclusions

The combination of NSAIDs with paracetamol or metamizole is a well-established practice in the clinical setting. Despite the clear advantages of using analgesic combinations, demanding investigation and regulatory considerations make the development of new parenteral non-opioid analgesics FDCs a very complex task. The development of several parenteral ready-to-use FDCs is conceptually possible; however, it is necessary to perform well-designed RCTs to produce solid clinical evidence and make market predictions to assess the viability of the introduction of new parenteral ready-to-use FDCs.

## Data Availability

Not applicable.

## Abbreviations

AAPM	American Academy of Pain Medicine
AM404	N-(4-Hydroxyphenyl) arachidonylamide
API	Active pharmaceutical ingredient
ASA	American Society of Anaesthesiologists
ATP	Adenosine triphosphate
b.i.d	Two times a day
CB1	Cannabinoid receptor type 1
CHMP	Committee for Medicinal Products for Human Use
CNS	Central nervous system
COX	Cyclooxygenase
ERAS	Enhanced recovery after surgery
FDA	Food and Drugs Administration
FDC	Fixed-dose combination
IM	Intramuscular
IV	Intravenous
MSF	Doctors Without Borders
NAPQI	N-acetyl-p-benzoquinone imine
NMDA	N-Methyl-D-aspartic acid
NOA	Non-opioid analgesic
NRS	Numeric rating scale
NSAID	Non-steroidal anti-inflammatory drug
o.d	Once a day
OTA	Orthopaedic Trauma Association
OTC	Over-the-counter
PGE2	Prostaglandin E2
q.i.d	Four times a day
PID	Pain intensity difference
PPTT	Pressure pain tolerance threshold
SC	Subcutaneous
SPC	Summary of product characteristics
SPID	Sum of pain intensity difference
SSRI	Selective serotonin reuptake inhibitor
t.i.d	Three times a day
TRPV1	Transient receptor potential vanilloid type 1
US	United States
USA	United States of America
VAS	Visual analog scale
VRS	Verbal rating scale
